# Mechanisms of *Ardisia japonica* in the Treatment of Hepatic Injury in Rats Based on LC-MS Metabolomics

**DOI:** 10.3390/metabo12100981

**Published:** 2022-10-17

**Authors:** Tian Fu, Shuiling Qin, Huajuan He, Kefeng Zhang, Wei Zhang, Xin Tang, Wei Wu

**Affiliations:** 1 School of Pharmacy, Guilin Medical University, Guilin 541199, China; 2 Pharmacology Laboratory of Prevention and Treatment of High Incidence of Disease, Guilin Medical University, Guilin 541199, China; 3 School of Public Health, Guilin Medical University, Guilin 541199, China

**Keywords:** *A. japonica*, immune liver injury, non-targeted metabolomics, metabolic pathway analysis

## Abstract

The mechanism of action of *Ardisia japonica* in the treatment of immune liver injury was systematically analyzed from the perspective of the biological metabolic network by using non-targeted metabolomics combined with biological network analysis tools. A rat model of acute immune hepatic injury was established by Concanavalin A (Con A) and the efficacy of the treatment of acute immune liver injury was judged by gavage of *A. japonica*. Liquid chromatography-mass spectrometry (LC-MS)-based plasma metabolomics was used to identify the key metabolites and metabolic pathways for the hepatoprotective effects of *A. japonica*. The results demonstrated that *A. japonica* reduced the levels of inflammatory parameters, decreased hepatic malondialdehyde levels, and enhanced hepatic antioxidant enzyme activity in animal experiments. The clustering of metabolomic samples showed significant separation in principal component analysis plots and the three groups in PLS-DA and OPLS-DA models could be clearly distinguished in multivariate statistical analysis. Among the 937 total metabolites, 445 metabolites were significantly different between the control and model groups, while 144 metabolites were identified as metabolites with differences between the model and administration groups, and a total of 39 differential metabolites were identified to affect the metabolic levels of the three groups. The differential metabolites were principally involved in the citric acid cycle, glutathione metabolism, vitamin B6 metabolism, and steroid hormone biosynthesis. This study found that *A. japonica* can significantly inhibit acute liver injury in rats, and exert a hepatoprotective effect through anti-inflammatory effect, inhibition of lipid peroxidation, improvement of the antioxidant defense system, and regulation of metabolites and related metabolic pathways. This study will provide a theoretical basis for the application of *A. japonica* in the treatment of the liver injury.

## 1. Introduction

Autoimmune hepatitis (AIH) is an inflammation of the liver parenchyma mediated by autoimmune reactions against hepatocytes, characterized by positive serum autoantibodies, hyper immunoglobulin G (IgG) and γ-globulinemia, and interface inflammation in liver histology, which, if left untreated, can often lead to cirrhosis and liver failure [1]. AIH has become one of the most prevalent liver diseases worldwide [2], and the pathogenesis of AIH is not yet well understood. Nowadays, AIH is diagnosed by the observation of transaminase levels, IgG levels, serum antinuclear antibodies, and liver histopathology [3]. However, the available methods are performed after the onset of the lesion, and there is no sensitive and accurate method for the early clinical diagnosis of acute immune liver injury. Metabolomics provides a new method for pre-morbid diagnosis and drug therapy monitoring of acute immune liver injury.

*A. japonica*, known as Japanese *Ardisia* or marlberry, is used as a medicinal plant in traditional Chinese medicine (TCM) [4]. The pharmacodynamic components of *A. japonica* are saponins, coumarins, benzoquinones, and flavonoids [4,5,6]. It has pharmacological activities such as relieving cough and asthma, protecting the liver, and anti-inflammatory, anti-viral, and anti-tumor activities [7,8]. In southern China, *A. japonica* is commonly used in folk to treat chronic hepatitis and has achieved a good therapeutic effect [9]. However, its pharmacodynamic material basis and mechanism of action are still unclear.

Metabolomics is an emerging platform for understanding the entire spectrum of endogenous metabolites in integrated biological systems and their dynamics in response to endogenous and exogenous factors [10]. The holistic view of metabolomics is fully consistent with the holistic thinking of TCM and the integrative effects of herbal medicine [11]. Thus, metabolomics offers a new and promising approach for assessing the therapeutic effects of herbal medicines and elucidating their underlying mechanisms [12,13,14]. However, there have been no reports on the use of metabolomic approaches to study the hepatoprotective effects of *A. japonica*. Liquid chromatography-mass spectrometry (LC-MS) provides excellent separation efficiency and high detection sensitivity and has become a common analytical platform for metabolomic studies [15]. Therefore, it is of great importance to perform the analysis of Chinese herbal medicine *A. japonica* by LC-MS metabolomics combined with a network analysis platform.

In this study, the rat model of acute immune liver injury induced by Con A was used, based on LC-MS plasma metabolomics, the changes of endogenous substances in the model rat induced by *A. japonica* were investigated, and the therapeutic material basis and mechanism of action of *A. japonica* were revealed.

## 2. Materials and Methods

### 2.1. Experimental Animals

SPF SD (Sprague-Dawley) male rats, weighing 200 ± 5 g, were purchased from Hunan Slaughter Jingda Laboratory Animal Co. The room temperature was maintained at 25 ± 0.5 °C and the humidity at 55 ± 5%, while the alternating light and dark cycles were maintained at 12 h/12 h. Animal Certificate No. 430727220100813863.

### 2.2. Preparation of Aqueous Extracts of A. japonica

The whole herb of *A. japonica* was ground into a coarse powder and stored in a dry and cool place. A certain amount (300 g) of coarse powder was weighed, soaked in 10 times the amount of water overnight, and then decocted twice, 1.5 h each time. The decoctions were mixed and concentrated to 1 g/mL, the concentrate was centrifuged at 5000 rpm for 15 min, and the supernatant was taken and concentrated to 1.5 g/mL. The obtained concentrate was then refrigerated at 4 °C.

### 2.3. Grouping, Modeling and Administration of Experimental Animals

SPF grade rats were randomly divided into the control group, model group, *A. japonica* extracts low dose group, *A. japonica* extracts high dose group, and positive control (Bifendate, BFD) group (Beijing Xiehe Pharmaceutical Co., Beijing, China) for three days of adaptive feeding, with six rats in each group. The control and model groups were given saline, and the remaining groups were given the drug once a day for 10 days. At 1 h after the final dose, Con A (Biosharp) 25 mg/kg was given in the tail vein of all groups except the control group, which was given an equal volume of saline. After 12 h, the blood sample was obtained from the aorta and subsequently centrifuged at 3000 rpm for 10 min at 4 °C. The collected supernatant was transferred to EP tubes for immediate freezing and then stored at −80 °C and thawed before analysis. Liver tissue was removed, and one portion was stored in liquid nitrogen and one portion was fixed in 4% paraformaldehyde (Solarbio, Beijing, China) solution for HE pathology sections. The animal experiments of the project met the requirements of animal welfare ethics and passed the animal welfare ethics investigation by IACUC of Guilin Medical University. No: GLMC-IACUC-2022012.

### 2.4. Detection of Biochemical Indicators and Oxidative Markers in Hepatocytes

The kits purchased from Nanjing Jiancheng Institute of Biological Engineering were used, and the alanine transaminase (ALT) (Cat.No.C009-2-1) and aspartate transaminase (AST) (Cat.No.C010-1-1) measurements were performed according to the instructions provided by the kit supplier. Liver tissues were assayed for malondialdehyde (MDA) (Cat.No. A003-1-2) content and superoxide dismutase (SOD) (Cat.No. A001-3-2) activity levels according to the instructions provided [16].

### 2.5. Metabolites Extraction from Plasma

Prior to LC-MS analysis, all cryopreserved plasma was pretreated. First, the plasma samples were slowly thawed on ice, 100 μL of the sample was placed in an EP tube, and 400 μL of 80% aqueous methanol (Thermo Fisher, Waltham, MA, USA) was added. The sample was then vortex-shocked, left to stand in an ice bath for 5 min, and centrifuged at 15,000× *g* for 20 min at 4 °C; an amount (100 μL) of supernatant was aspirated and diluted with mass spectrometry grade water to 53% methanol [17]. Then, the sample was centrifuged at 15,000× *g* for 20 min at 4 °C [18,19]. The supernatant was collected and analyzed by LC-MS [20]. The supernatant of each test sample was mixed with 10 μL of each sample and used as quality control (QC) samples, and one QC sample was inserted for every five samples to check the stability of the instrument.

### 2.6. LC-MS Analysis

Instruments: Q Exactive™ HF-X (Thermo Fisher, Waltham, MA, USA), Vanquish UHPLC (Thermo Fisher, Waltham, MA, USA); Chromatographic columns: Hypesil Gold column (C18) (Thermo Fisher, Waltham, MA, USA). The column temperature was 40 °C, and the flow rate was 0.2 mL/min. In positive mode: mobile phase A was 0.1% formic acid (Thermo Fisher, USA); mobile phase B was methanol (Thermo Fisher, Waltham, MA, USA). In negative mode: mobile phase A was pH 9.0, 5 mM ammonium acetate (Thermo Fisher, Waltham, MA, USA); mobile phase B was methanol.

The scan range was *m/z* 100–1500, spray voltage was 3.5 kV; sheath gas flow rate was 35 psi; Aux gas flow rate is 10 L/min; Capillary temp was 320 °C; S-lens RF level was 60; Aux gas heater temp was 350 °C. Mobile phase gradient settings: Time: 0–1.5 min, A: 98%, B: 2%; Time: 3 min, A: 15%, B: 85%; Time: 10 min, A: 0, B: 100%; Time: 10.1–12 min, A: 98%, B: 2%.

### 2.7. Data Processing and Statistical Analysis

The raw data were preprocessed by using CD 3.1 data (Thermo Fisher, Waltham, MA, USA) processing software. Subsequently, peak extraction was performed based on the set ppm, signal-to-noise ratio (S/N), adduct ions and other information, and the peak area was quantified. Multidimensional statistical analysis (PCA, PLS-DA and OPLS-DA) was performed by using SIMCA-P. The potential biomarkers were screened according to the criteria of variable projection importance (VIP) > 1.0 and *p* < 0.05, and their structures were identified by HMDB database (https://hmdb.ca/metabolites (accessed on 6 June 2022), MassBank (https://massbank.eu/MassBank/Search (accessed on 6 June 2022) and LIPID Maps database (http://www.lipidmaps.org/ (accessed on 6 June 2022) and other databases. The identified biomarkers were imported into the MetaboAnalyst 5.0 database (https://www.metaboanalyst.ca/ (accessed on 6 June 2022) and metabolic pathway analysis was performed based on the KEGG database (https://www.genome.jp/kegg/pathway.html (accessed on 6 June 2022) to reveal the key metabolic pathways.

GraphPad Prism 8.0.2 software (GraphPad, San Diego, CA, USA) was used for data plotting, and the data were expressed as mean ± SEM. SPSS 26.0 software (SPSS, IBM, New York, NY, USA) was used for statistical analysis, the statistical differences between two groups were compared by *t*-test, and the statistical differences between multiple groups were compared by one-way ANOVA. Statistical significance was defined as *p* < 0.05.

## 3. Results

### 3.1. Therapeutic Effect of A. japonica on Con A-Induced Immune Liver Injury

**Figure 1:**
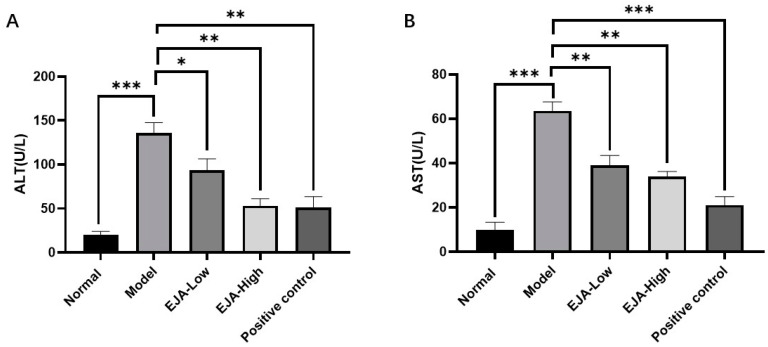
*A. japonica* treatment attenuated Con A-induced acute liver injury. (**A**) Effect of EJA on serum ALT levels; (**B**) Effect of EJA on serum AST levels. (ns = nonsignificant, * *p* < 0.05, ** *p* < 0.01, and *** *p* < 0.001 among the compared groups. Student *t*-test was performed. EJA: *A. japonica* extract).

**Figure 2:**
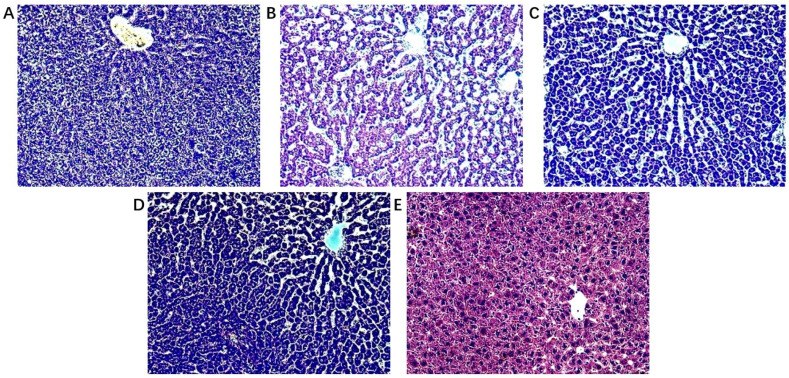
Histopathological observation (**A**) Control group; (**B**) Model group; (**C**) *A. japonica* low dose group; (**D**) *A. japonica* high dose group; and (**E**) Positive control group (Bifendate, BFD). Scale bar = 200 µm.

The biochemical indicators of the model group changed significantly compared to control and treated groups (Figure 1). The detection of serum markers of liver injury (ALT and AST) showed that Con A-induced acute liver injury resulted in a significant increase of ALT and AST levels, which was significantly alleviated by the treatment with *A. japonica* in a dose-dependent manner. In addition, HE staining of liver tissues showed that there was a large infiltration of inflammatory cells in the model group compared with the control group, and there was a large amount of vacuolar-like degeneration of hepatocytes. After being treated with *A. japonica*, liver tissue damage was alleviated, and the therapeutic effect of a high dose was more obvious, which could significantly alleviate the Con A-induced hepatic sinusoidal congestion, liver tissue inflammation, and hepatocyte necrosis in rats (Figure 2).

### 3.2. Alleviating Oxidative Stress Induced by Acute Liver Injury

**Figure 3:**
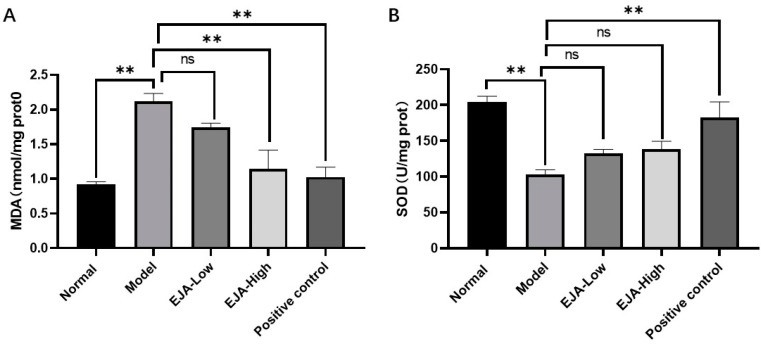
*A. japonica* alleviates the level of oxidative stress induced by acute liver injury. (**A**) MDA levels in rat liver tissues; (**B**) SOD activity in rat liver tissues, Student’s *t*-test was performed (ns: not significant, ** *p* < 0.01).

Compared with the control group, the level of MDA in the liver homogenate of the model group was significantly increased and the activity of SOD was significantly reduced. Compared with the model group, the level of MDA in the treatment group was decreased in a dose-dependent manner, and the high and low doses of *A. japonica* aqueous extract had a mitigating effect on the decrease in SOD activity levels (Figure 3).

### 3.3. Stability of Equipment

Ion chromatograms of rat plasma samples were obtained through LC-MS in both positive and negative ion modes. The unsupervised principal component analysis (PCA) was used to cluster the QC samples to examine the stability of the instrumentation system. The results are shown in Supplementary Materials Figure S1. The groups were clearly separated in the unsupervised PCA score plot, and the distribution of QC samples was clustered together. This indicates that the general method is stable and the quality of the data is high.

### 3.4. Multivariate Statistical Analysis

#### 3.4.1. PCA and PLS-DA Analysis

**Figure 4:**
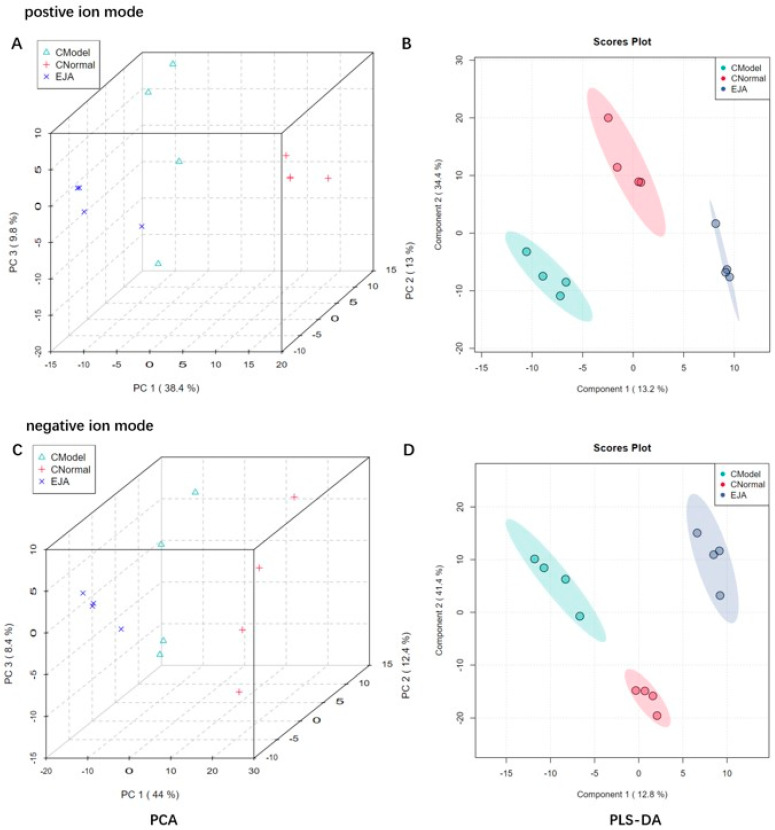
Discrimination of plasma metabolic profiles in normal group (CNormal), model group (CModel), and EJA administered group. Plots of PCA scores (**A**) and PLS-DA scores; (**B**) in positive ion mode; Plots of PCA scores; (**C**) PLS-DA scores; and (**D**) in negative ion mode.

To obtain sufficient metabolite information, plasma samples from the normal group, the model group, and the *A. japonica* extract (EJA) group were analyzed in positive and negative ion modes. As shown in Figure 4A,C, the PCA score plot shows the distribution between the three groups in 3D space. Samples from the same group are almost clustered together and within the 95% confidence interval, the variables observed in the samples are confirmed to be biologically relevant.

Partial least squares discriminant analysis (PLS-DA) was performed on rat plasma samples. Significant clustering was observed among the normal, model, and EJA groups in both positive and negative ion modes, indicating that inter-group differences were more significant than individual differences (Figure 4B,C). In addition, the model group could be significantly separated from the normal group in the positive and negative ion mode, indicating that significant metabolic changes occurred after modeling, and there were differential metabolites between the two groups, suggesting that Con A could induce liver metabolic disorders. The EJA group showed a tendency to regress to the normal control group compared to the model group, indicating that EJA can reverse the metabolic disorders induced by Con A.

#### 3.4.2. Orthogonal PLS-DA Analysis

**Figure 5:**
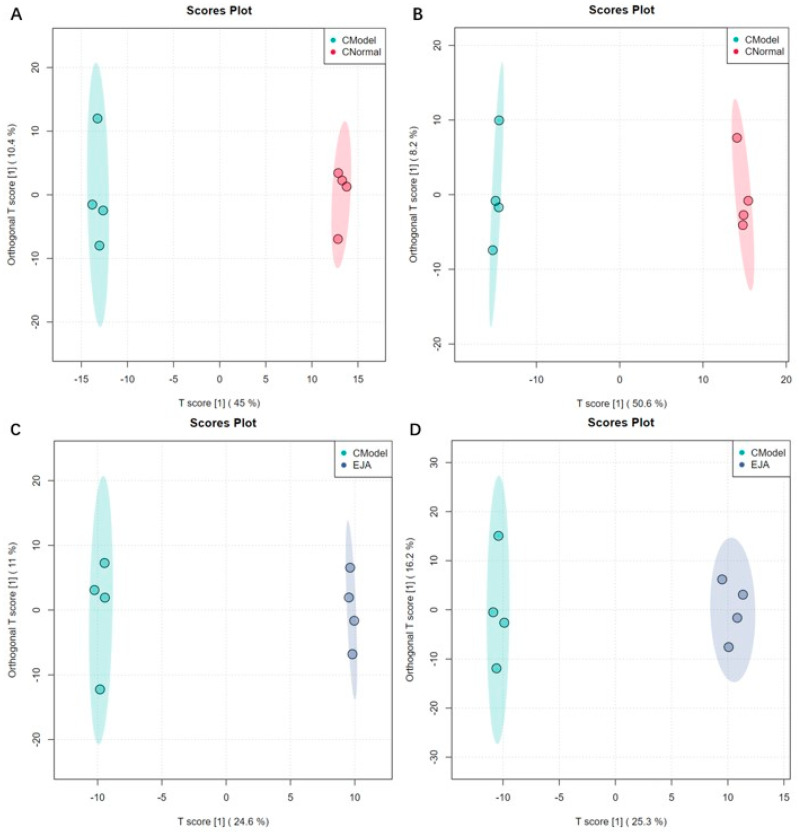
Sample distribution of different groups in OPLS-DA mode. (**A**) the normal group and the model group in positive ion mode; (**B**) the normal group and the model group in negative ion mode; (**C**) the model group and the treatment group in positive ion mode; and (**D**) the model group and the treatment group in negative ion mode.

Orthogonal PLS-DA (OPLS-DA) is a supervised discriminant analysis statistical method, which can improve classification and discrimination capacity, and reduce the influence of interference factors [21]. To further investigate the therapeutic mechanism of *A. japonica*, OPLS-DA was performed to identify significantly varying ions between the normal, model, and treatment groups. As shown in Figure 5, the normal group, the model group, and the treatment group could be significantly distinguished from each other in both positive and negative ion modes. For the normal group and the model group, R2Y = 0.999 and Q2 = 0.966 in positive ion mode; R2Y = 0.999 and Q2 = 0.967 in negative ion mode. For the model group and the treatment group, R2Y = 0.999 and Q2 = 0.823 in positive ion mode; R2Y = 0.997 and Q2 = 0.812 in negative ion mode (Supplementary Materials Figure S2). The results showed that the model had a good fit and predictive capability, with no over-fit, and could be used to screen differential markers.

**Figure 6:**
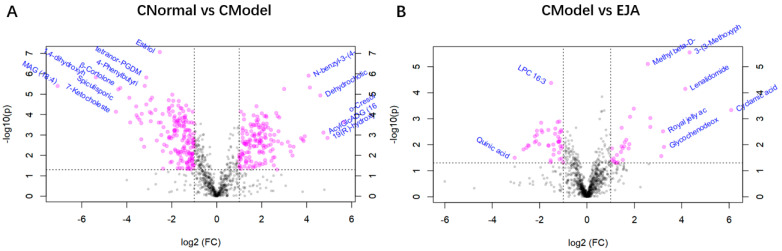
Volcano plot of multiplier variation. (**A**) Volcano plot of change in the difference between control and model groups; (**B**) Volcano plot of the difference between the model group and the drug administration group. Note: Horizontal coordinates are multiples of change, vertical coordinates are *t*-test *p*-values.

Volcano plots show trends in differential metabolite expression in the control versus model groups and in the model versus administered groups (Figure 6). Each dot represents a specific metabolite. The left side of the origin refers to the down-regulated metabolites and the right side of the origin refers to the up-regulated metabolites.

### 3.5. Screening and Identification of Potential Biomarkers

**Figure 7:**
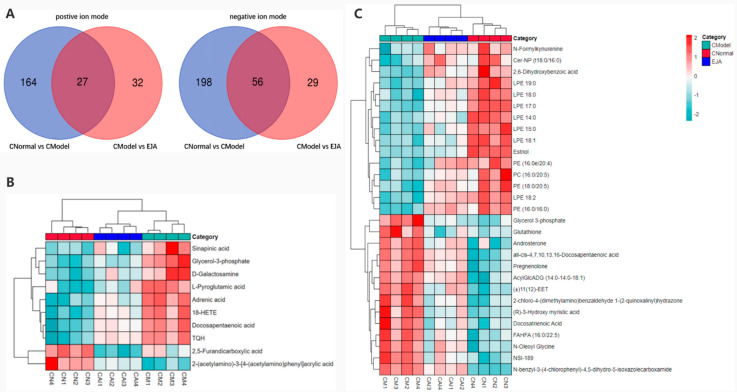
(**A**) Venn diagram showing the overlapping metabolites in different groups; (**B**) differential metabolite correlation thermal images in positive ion mode; and (**C**) differential metabolite correlation thermal images in negative ion mode.

In positive and negative ion modes, 589 variable ions were screened from the normal vs. model group, and the model vs. EJA group, respectively. (Figure 7). As visualized by the Venn diagram, 191 metabolite abundances were identified to change in the model group compared to the control group in the positive ion mode, while 254 metabolites changed significantly in the negative ion mode. After the *A. japonica* administration, plasma levels of all 27 metabolites were regulated to normal levels in the positive ion mode, while 56 metabolites were regulated to normal plasma levels in the negative ion mode (Figure 7A).

To study the effect of *A. japonica* on endogenous substance metabolism in rats, inter-group differences were analyzed using the OPLS-DA method. Potential metabolites that contributed significantly to clustering and differentiation were selected based on their Variable Importance Projection (*VIP*) values and *p*-values. Metabolites with *VIP* > 1 and *p* < 0.05 were chosen as potential differential metabolites to evaluate the mechanism of *A. japonica* in treating acute immune liver injury induced by Con A. According to the screening criteria, 39 differential metabolites were identified. (Table 1).

To investigate the effect of *A. japonica* on Con A-induced disordered metabolites, Heatmaps were drawn according to the relative intensities of 39 metabolites in the normal, model, and EJA administered groups to visualize the relative amounts of potential biomarkers in each sample (Figure 7). It can be seen that 16 metabolites were up-regulated in the model group compared to the control group, while the other 23 metabolites were inhibited.

### 3.6. Differential Metabolic Pathway Analysis

**Figure 8:**
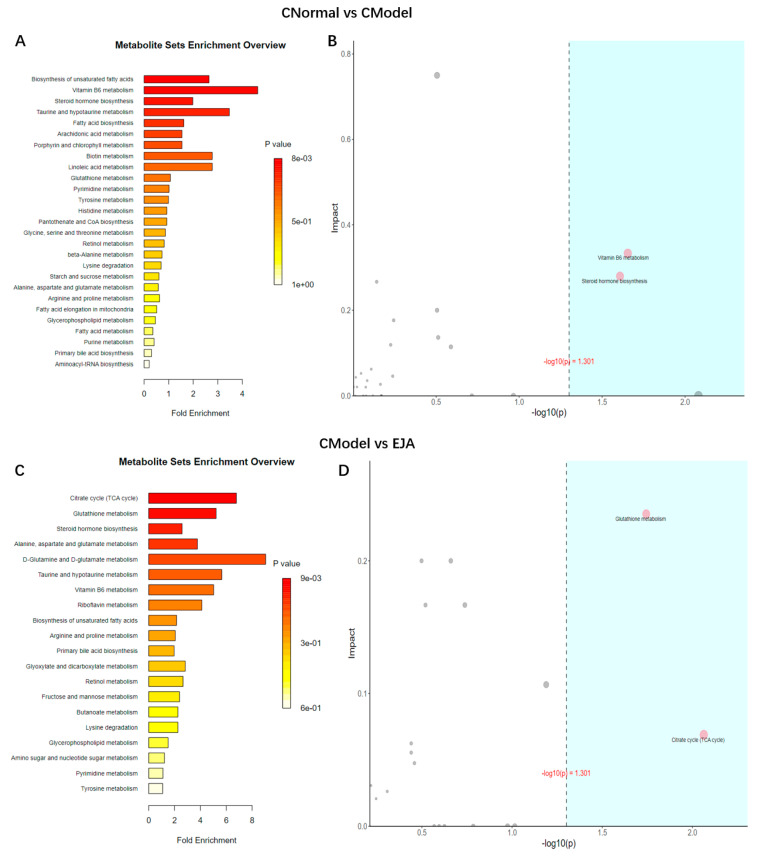
Metabolic pathway analysis. (**A**) Enrichment analysis of normal group and model group. (**B**) Topology analysis of normal group and model group. (**C**) Enrichment analysis of model group and drug administration group. (**D**) Topological analysis of model group and drug administration group. Note: The horizontal coordinates of B and D are the ORA analysis *p*-values, with the blue area being significant (*p* < 0.05); the vertical coordinates are the topological analysis effects.

Significantly different metabolites were selected, and Over-Representation Analysis (ORA) was used to find the KEGG pathways that were significantly enriched in these metabolites and calculated the topological influence. For the significantly enriched KEGG pathways, metabolic pathway diagrams were drawn and metabolites with significant differences were marked in the pathway diagrams. The results showed that the differences in metabolic pathways between the control group and the model group included unsaturated fatty acid biosynthesis, vitamin B6 metabolism, steroid hormone biosynthesis, taurine, and hypotaurine metabolism (Figure 8A). The most relevant metabolic pathways are vitamin B6 metabolism and steroid hormone biosynthesis (Figure 8B). The differences in metabolic pathways between the model group and EJA group included citric acid cycle (TCA cycle), glutathione metabolism, steroid hormone biosynthesis, alanine, aspartate, and glutamate metabolism (Figure 8C), among which the TCA cycle and glutathione metabolism are the most important (Figure 8D).

## 4. Discussion

Autoimmune hepatitis is a chronic progressive liver disease with complicated pathological mechanisms [22]. The effect of *A. japonica* treatment on immune liver injury has not been previously reported in the literature. In the present study, a Con A-induced immunological liver injury model was established to explore the hepatoprotective effects of *A. japonica.* LC-MS metabolomics analysis was used to reveal the disordered metabolites in acute immunological liver injury and metabolites in response to *A. japonica* treatment under this condition.

ALT and AST are reliable markers for detecting acute immune liver injury [23]. Liver function tests typically show a hepatocellular pattern of injury, with an increase in aminotransferase-es, that can be mildly elevated or up to 50 times the upper normal value. Alanine aminotransferase (ALT) is typically higher than Aspartate aminotransferase (AST). Cholestatic enzymes are usually normal or mildly elevated unless there is an overlap with primary biliary cholangitis (PBC) or primary sclerosing cholangitis (PSC) [24,25,26,27,28,29,30]. In this study, acute immune liver injury in rats was induced by Con A, in which the elevated biochemical levels of ALT and AST indicated the successful modeling of acute immune liver injury. It was found that *A. japonica* could improve liver function by modulating AST and ALT in the acute immune liver injury model, suggesting that *A. japonica* has both therapeutic and preventive effects on immune liver injury. MDA, a secondary metabolite produced by free-radical attack, is widely used to reflect the extent of cellular injury [31]. At the same time, *A. japonica*. reduced hepatic malondialdehyde (MDA) levels and enhanced hepatic antioxidant enzyme activity, suggesting that *A. japonica* exerts hepatoprotective effects through anti-inflammatory effects and inhibits lipid peroxidation, and improves the antioxidant defense system. The typical aspect of AIH is that of a severe chronic hepatitis, with intense portal and lobular inflammation, severe interface hepatitis, and hepatocyte damage [32]. Histopathological sections showed significant congestion of liver tissue after Con A modelling, while a small infiltration of inflammatory cells could be seen, indicating successful modelling of immune liver injury caused by knife bean protein A. Pre-administration of the drug significantly reduced liver tissue damage, hepatic sinusoidal congestion, liver tissue inflammation, and hepatocyte necrosis. The results showed that the mechanism of Con A-induced acute immune liver injury was related to lipid peroxidation and anti-oxidation defense mechanisms, and *A. japonica* had a definite therapeutic effect on immune liver injury.

Autoimmune liver injury is often associated with metabolic disturbances due to changes in the metabolite profile [33,34]. Several metabolomic studies have shown dramatic changes in the liver and blood metabolite profiles in acute immune liver injury [35,36]. Our animal experiments have confirmed that *A. japonica* can effectively alleviate immune liver injury and has a hepatoprotective. Based on this, LC-MS metabolomics technology [37,38] was used to study the metabolite changes in liver injury rats after administration of *A. japonica* and investigate the significantly enriched metabolic pathways. Calculation of the Pearson correlation coefficient between QC samples based on the relative quantitative values of the metabolites [39]. The 95% confidence interval of QC samples indicated that the chromatographic separation and mass spectrometry collection of biological samples were relatively stable throughout the analysis process, and the accuracy and reliability of data were good. In both positive and negative ion modes, there was a dispersion tendency between groups and aggregation within groups, indicating that the difference between groups was greater than that within groups. There was a tendency of dispersion between the model group and the normal group, suggesting that the model rats had obvious metabolic disorders induced by Con A. Compared with the model group, the *A. japonica* administration group showed a discrete tendency, and converged with the blank group, which proved that *A. japonica* could regulate the aforementioned metabolic disorder. Compared with the normal group, 39 endogenous metabolites were significantly different in the plasma samples of the model group, and plasma metabolite levels in rats tended to be normal after *A. japonica* treatment. It is speculated that *A. japonica* exerts hepatoprotective effects against Con A-induced liver damage by regulating the metabolic pathways of the TCA cycle, glutathione metabolism, steroid hormone biosynthesis, alanine, aspartate, and glutamate metabolism. *A. japonica* has largely corrected these metabolic disturbances, which indicates that *A. japonica* achieves a therapeutic effect by regulating specific metabolites. These results suggest that *A. japonica* exerts a therapeutic effect on acute immune liver injury by altering these biomarkers and related metabolic pathways (Supplementary Materials Figures S3 and S4).

At present, there is no reasonable explanation for the metabolic pathways involved in the pathogenesis of acute immune liver injury. This study demonstrated that the altered metabolic pathways associated with Con A-induced acute immune liver injury could be reversed by *A. japonica*, the most important of which are vitamin B6 metabolism and the TCA cycle. Studies have confirmed that “vitamin B6 metabolism” is impaired in alcoholic liver disease and that acute alcohol-induced hepatic lipid deposition can be reduced by supplementation with vitamin B6 [40]. Vitamin B6 is also found to be involved in both cellular and humoral immunity during the immune response. Vitamin B6 deficiency directly leads to atrophy of human lymphocyte organs, a reduction in the number of lymphocyte genes produced, a significant reduction in human immunity, altered responses to antibodies, and indirectly a reduction in the number of genes produced about interleukin-2 (IL-2) [41,42].

The TCA cycle is a metabolic pathway for sugars, lipids, and amino acids [43], which is influenced by some rate-limiting enzymes [44]. MDH can catalyze the reversible conversion of malate to oxaloacetate using NAD+ or NADP+ as cofactors [45]. The key enzymes of the TCA cycle play an important role in regulating the energy of the body [43,44,45,46]. It has been shown that inflammation disrupts the TCA cycle, and metabolic intermediates of the TCA cycle have been shown to play an important role in regulating the intrinsic immune cell response [47,48], leading to remodeling of the TCA cycle and accumulation of intracellular metabolic intermediates [49,50], resulting in mitochondrial dysfunction [50]. In the event of disruption of the TCA cycle, citric acid acts in reverse in the reversible reaction of the TCA cycle, which is important not only for lipid biosynthesis in macrophages and dendritic cells, but also for the production of pro-inflammatory and anti-inflammatory mediators [51,52,53]. Citric acid links many important cellular processes such as linking sugar, lipid metabolism, and protein modification. Citric acid decarboxylates isocitric acid to produce OGDH via IDH. citric acid can be transported from the mitochondria to the cytoplasm via the mitochondrial citric acid carrier in exchange for malic acid [54], suggesting that the expression of key enzymes in the mitochondrial TCA cycle is affected in the inflammatory state and that the impairment of key enzymes in the TCA cycle is somewhat improved when inflammation is suppressed. This suggests that metabolites in the TCA cycle play a key role in the development of the inflammatory response. which is consistent with our findings. Therefore, our study suggests that multiple metabolic pathways are involved in Con A-induced liver injury and these metabolic disturbances can be reversed by treatment with *A. japonica*.

There are a number of limitations to our study. Firstly, the metabolomics data are large and complex. The number and type of metabolites identified in plasma may vary from cell to cell or tissue to tissue. Secondly, small samples were used in our study and there were only few metabolomic studies of TCM for AIH. In summary, this study is a preliminary exploration of the mechanisms of A. japonica through non-targeted metabolomics, and extensive studies are needed to explore deeper molecular mechanisms.

## 5. Conclusions

In conclusion, our study is the first to demonstrate the efficacy of *A. japonica* in the treatment of AIH. It was also demonstrated that *A. japonica* treatment reversed the changes in plasma metabolite abundance in AIH rats and indirectly activated the vitamin B6 and TCA circulating pathways, thereby restoring the metabolism of specific metabolites in plasma and ultimately ameliorating Con A-induced liver damage. This study identifies a group of metabolites that are activated in Con A-induced liver injury. Furthermore, *A. japonica* was shown to be effective in correcting these metabolic disturbances. The results obtained enhance the use of *A. japonica* as a candidate therapeutic agent for the prevention or treatment of Con A-induced acute liver injury. Our results provide a new biochemical mechanism by which *A. japonica* combats AIH and lays the foundation for the development of *A. japonica* as a clinical agent for the treatment of AIH.

## Figures and Tables

**Table 1:** Differential metabolites in plasma samples in positive and negative ion mode.

No.	ESI Mode	Metabolites	Formula	Molecular Weight	RT [min]	*m/z*	Normal vs. Model Trend	Model vs. EJA Trend
1	+	2,5-Furandicarboxylic acid	C_6_H_4_O_5_	156.0062	2.077	157.0135	Down	Up
2	+	L-Pyroglutamic acid	C_5_ H_7_ NO_3_	129.0428	2.152	130.0501	Up	Down
3	+	Docosapentaenoic acid	C_22_H_34_O_2_	330.256	10.248	331.2632	Up	Down
4	+	Sinapinic acid	C_11_H_12_O_5_	208.0945	1.393	247.0577	Up	Down
5	+	Adrenic acid	C_22_H_36_O_2_	332.2716	10.72	333.279	Up	Down
6	+	Glycerol-3-phosphate	C_3_ H_9_ O_6_ P	172.0139	1.408	173.0213	Up	Down
7	+	D-Galactosamine	C_6_H_14_ClNO_5_	215.0561	1.285	216.0633	Up	Down
8	+	2-(acetylamino)-3-[4-(acetylamino) phenyl] acrylic acid	C_13_H_14_N_2_O_4_	262.0957	5.447	263.1031	Down	Up
9	+	18-HETE	C_20_H_32_O_3_	302.2247	9.57	303.232	Down	Up
10	+	TQH	C_15_H_24_N_6_O_6_	384.1753	10.25	385.1826	Up	Down
11	−	®-3-Hydroxy myristic acid	C_14_H_28_O_3_	244.2034	8.453	243.1962	Up	Down
12	−	Estriol	C_18_H_24_O_3_	288.1755	7.509	287.1682	Down	Up
13	−	LPE 14:0	C_19_H_40_NO_7_P	425.2535	8.625	424.2463	Down	Up
14	−	Glutathione	C_10_H_17_N_3_O_6_S	307.0832	1.886	306.0759	Up	Down
15	−	LPE 17:0	C_22_H_46_NO_7_P	467.3004	9.89	466.2933	Down	Up
16	−	Pregnenolone	C_21_H_32_O_2_	316.2397	9.904	315.2325	Up	Down
17	−	FAHFA (16:0/22:5)	C_38_H_64_O_4_	584.4773	10.464	583.47	Up	Down
18	−	LPE 18:2	C_23_H_44_NO_7_P	477.2853	9.152	476.2779	Down	Up
19	−	LPE 18:1	C_23_H_46_NO_7_P	479.3009	9.69	478.2936	Down	Up
20	−	AcylGlcADG (14:0-14:0–18:1)	C_55_H_100_O_12_	952.7035	10.714	951.695	Up	Down
21	−	PC (16:0/20:5)	C_44_H_78_NO_8_P	825.5531	10.883	824.546	Down	Up
22	−	2-chloro-4-(dimethylamino) benzaldehyde 1-(2-quinoxalinyl) hydrazone	C_17_H_16_ClN_5_	325.1101	8.069	324.1027	Up	Down
23	−	(±)11(12)-EET	C_20_H_32_O_3_	320.2345	9.102	319.2272	Up	Down
24	−	NSI-189	C_22_H_30_N_4_O	366.2402	6.701	365.2329	Up	Down
25	−	PE (18:0/20:5)	C_43_H_76_NO_8_P	765.5305	10.867	764.5229	Down	Up
26	−	Docosatrienoic Acid	C_22_H_38_O_2_	334.2866	11.252	333.2794	Up	Down
27	−	LPE 18:0	C_23_H_48_NO_7_P	481.3168	10.295	480.3094	Down	Up
28	−	2,6-Dihydroxybenzoic acid	C_7_H_6_O_4_	154.0264	5.385	153.0191	Down	Up
29	−	N-benzyl-3-(4-chlorophenyl)-4,5-dihydro-5-isoxazolecarboxamide	C_17_H_15_ClN_2_O_2_	314.0816	7.23	313.0745	Up	Down
30	−	Cer-NP (t18:0/16:0)	C_34_H_69_NO_4_	601.5279	9.819	600.5206	Down	Up
31	−	N-Oleoyl Glycine	C_20_H_37_NO_3_	339.2771	9.906	338.2699	Up	Down
32	−	Androsterone	C_19_H_30_O_2_	290.224	9.733	289.2167	Up	Down
33	−	all-cis-4,7,10,13,16-Docosapentaenoic acid	C_22_H_34_O_2_	330.2558	10.256	329.2481	Up	Down
34	−	PE (16:0/16:0)	C_37_H_74_NO_8_P	691.5156	11.441	690.5097	Down	Up
35	−	PE (16:0e/20:4)	C_41_H_76_NO_7_P	725.5358	11.462	724.529	Down	Up
36	−	Glycerol 3-phosphate	C_3_H_9_O_6_P	172.0134	1.418	171.0061	Up	Down
37	−	LPE 15:0	C_20_H_42_NO_7_P	439.269	9.053	438.2615	Down	Up
38	−	LPE 19:0	C_24_H_50_NO_7_P	495.3317	10.563	494.3244	Down	Up
39	−	N-Formylkynurenine	C_11_H_12_N_2_O_4_	236.0792	5.186	235.072	Down	Up

## Data Availability

Data is available in the manuscript.

## Supplementary Materials

The following supporting information can be downloaded at: https://www.mdpi.com/article/10.3390/metabo12100981/s1, Figure S1: PCA analysis of the total sample; Figure S2: Distribution of test statistic; Figure S3: Analysis of vitamin B6 metabolic pathway; Figure S4: Analysis of TCA circulating metabolic pathways.
