# Supplementary material for: Mechanisms of Ardisia japonica in the Treatment of Hepatic Injury in Rats Based on LC-MS Metabolomics
Source: Metabolites. 2022 Oct 17;12(10):981. doi: 10.3390/metabo12100981 (PMC9610646; doi:10.3390/metabo12100981)
Supplement: Supplementary file 1 [file metabolites-12-00981-s001.zip › metabolites-1965380-supplementary.pdf]

metabolites

Mechanisms of *Ardisia japonica* in the Treatment of Hepatic Injury in Rats Based on LC-MS Metabolomics

Tian Fu [1,2,†], Shuiling Qin [1,2,†], Huajuan He [1], Kefeng Zhang [1,2], Wei Zhang [1], Xin Tang [3,*] and Wei Wu [1,2,*]

1 School of Pharmacy, Guilin Medical University, Guilin 541199, China
2 Pharmacology Laboratory of Prevention and Treatment of High Incidence of Disease, Guilin Medical University, Guilin 541199, China
3 School of Public Health, Guilin Medical University, Guilin 541199, China
* Correspondence: tangxin_glmc@126.com (X.T.); wuwei@glmc.edu.cn (W.W.)
† These authors contributed equally.

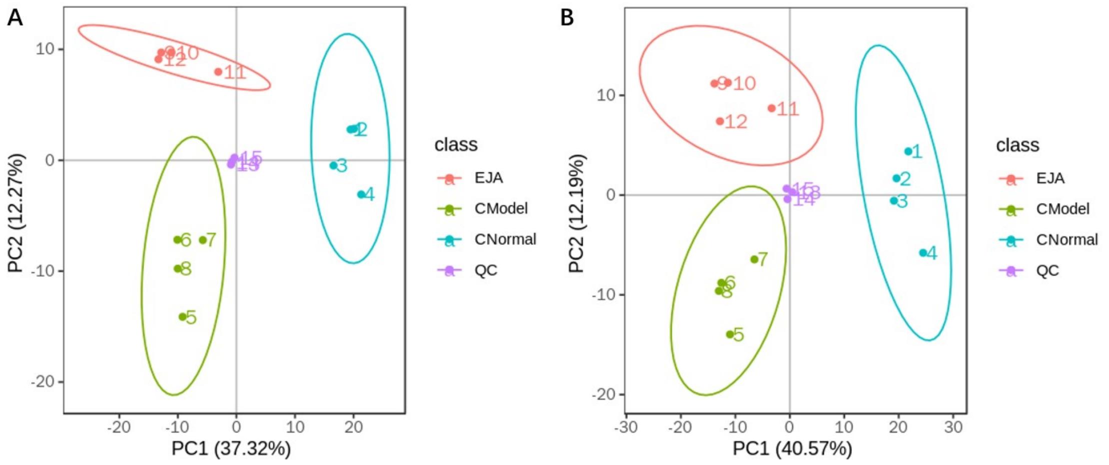

Supplementary materials Figure S1. PCA analysis of the total sample. (**A**) PCA plot of total samples in positive ion mode; (**B**) PCA plot of total samples in negative ion mode (Note: Scattered dots in different colors indicate samples of different experimental groups, and ellipses are 95% confidence intervals).

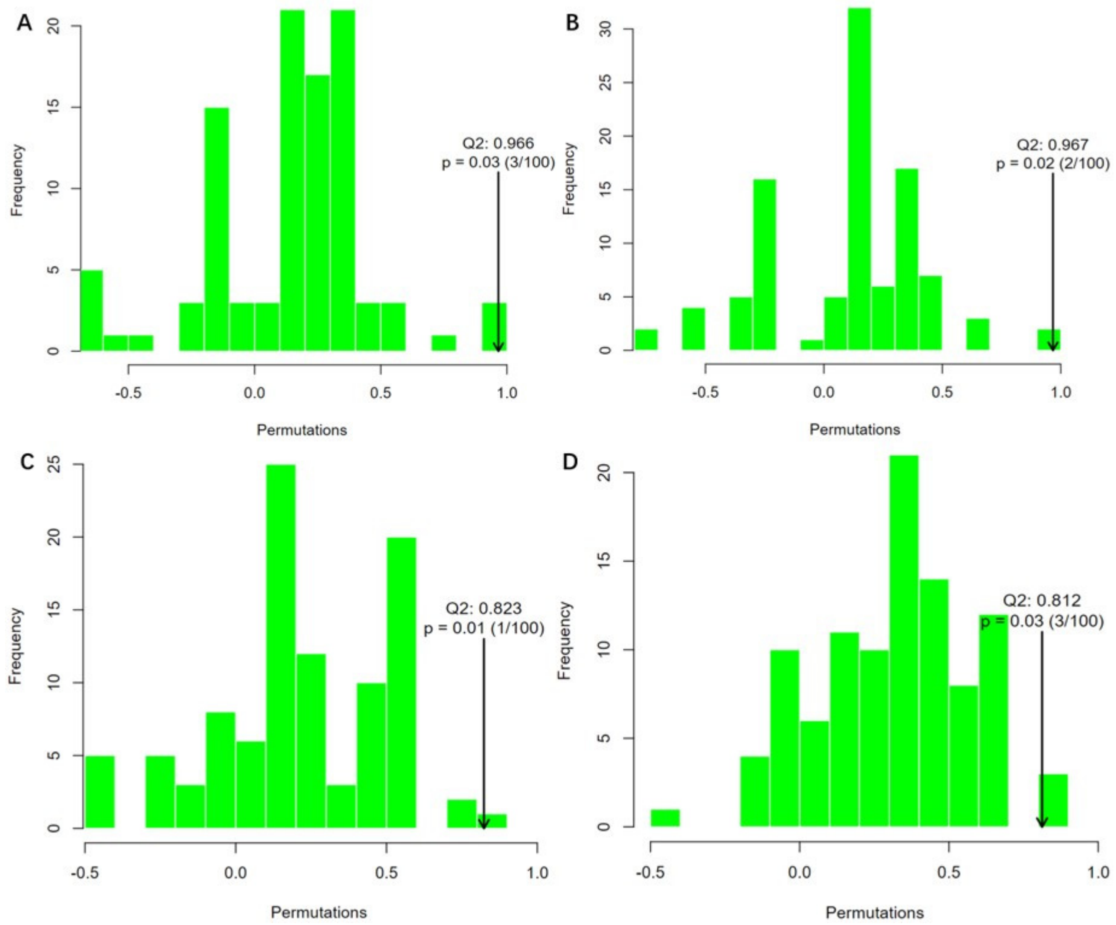

Supplementary materials Figure S2. Distribution of test statistic (Q2) and p-value of OPLS-DA permutation tests. Permutation test between normal and model groups in positive ion mode (**A**) and negative ion mode (**B**); Permutation test between model and treatment groups in positive ion mode (**C**) and negative ion mode (**D**). (Note: The distribution plots are the permutation random distribution of Q2, and the arrows point to the actual observed model Q2）.

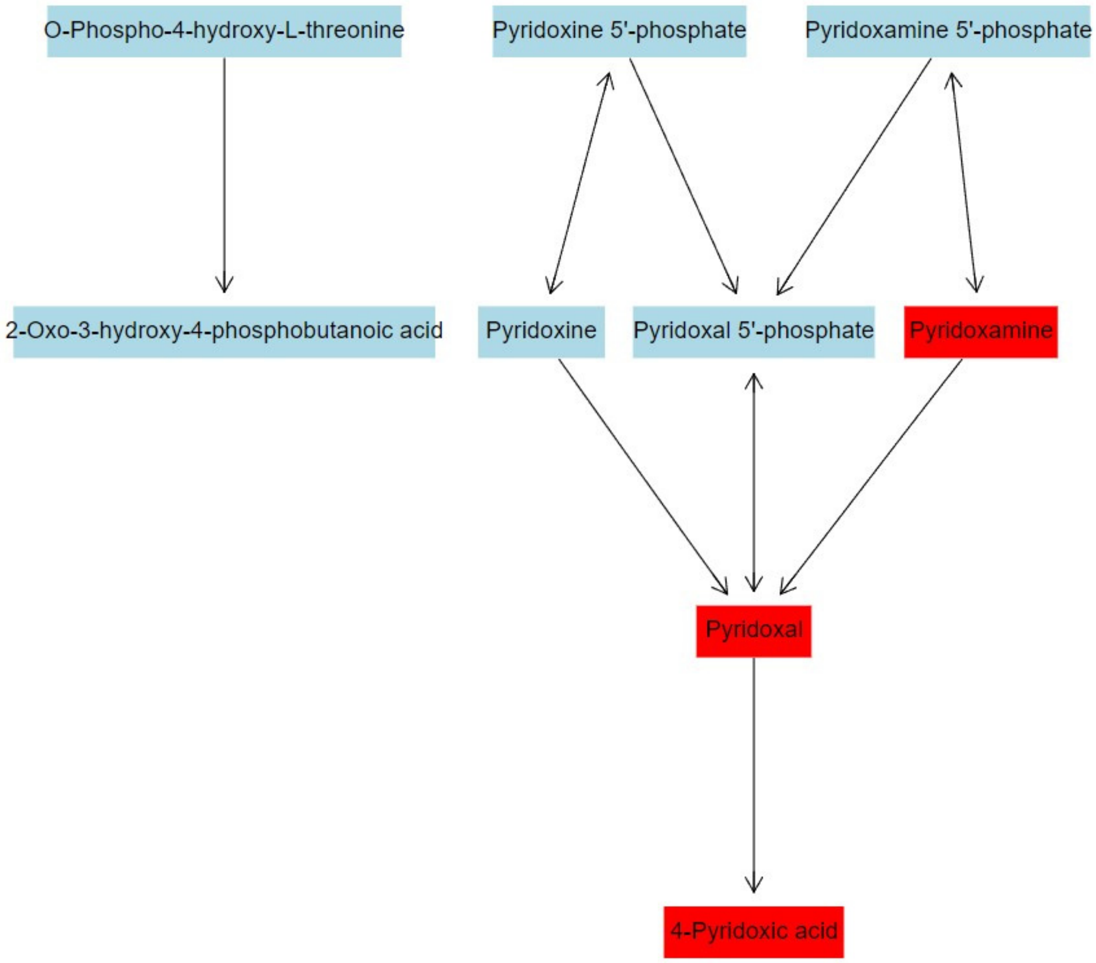

Supplementary materials Figure S3. Analysis of vitamin B6 metabolic pathway. (Note: Each arrow points from the substrate to the product, and the bidirectional arrows indicate that the reaction is reversible. Metabolites in red are those that differ significantly between groups).

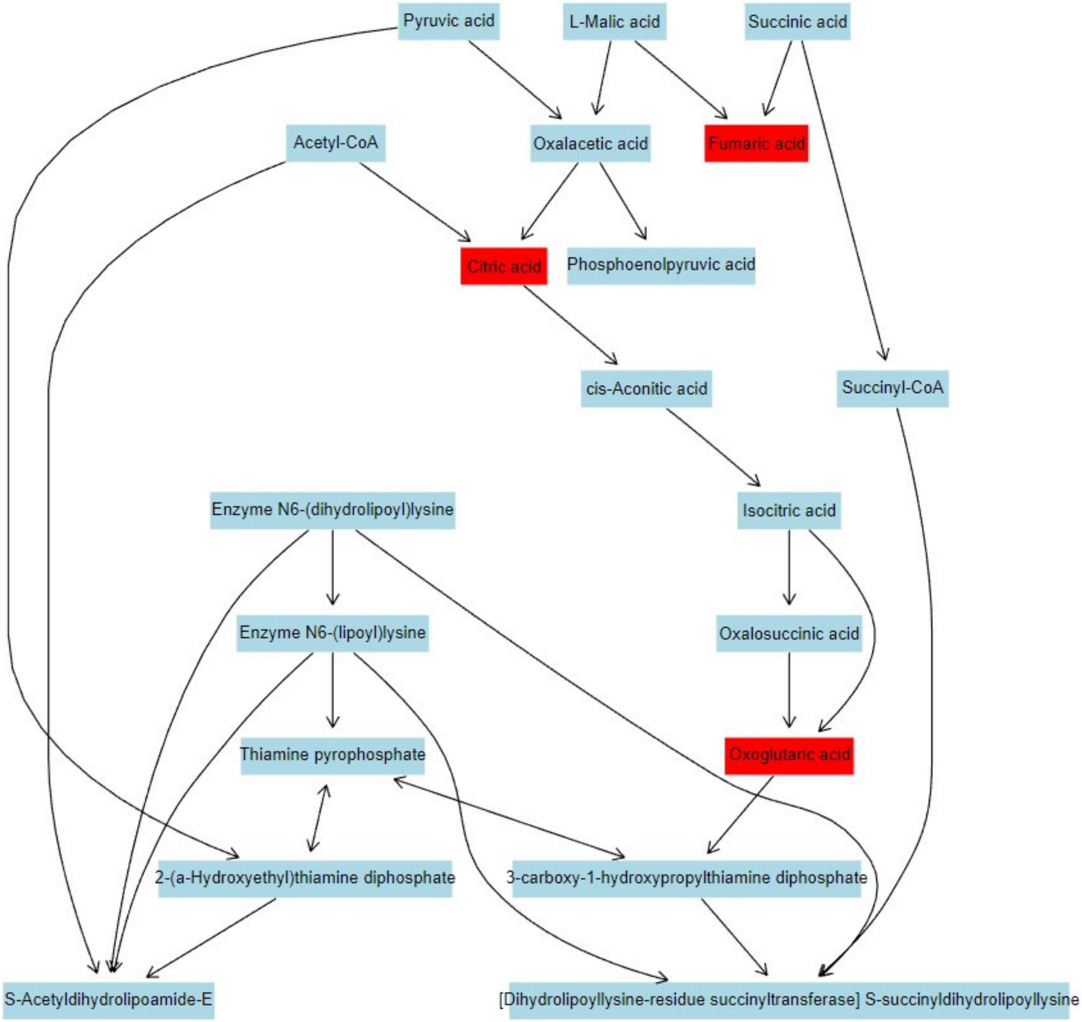

Supplementary materials Figure S4. Analysis of TCA circulating metabolic pathways. (Note: Each arrow points from the substrate to the product, and the bidirectional arrows indicate that the reaction is reversible. Metabolites in red are those that differ significantly between groups).
